# Phenotypic patterns in feline heart failure: A natural model for understanding variable disease severity in humans

**DOI:** 10.1016/j.isci.2026.116685

**Published:** 2026-07-10

**Authors:** Talitha C.F. Spanjersberg, Alma H. Hulsman, Guy C.M. Grinwis, Babette Janssen, C. Nina van der Wilt, Rogier J.A. Veltrop, Christian J.B. Snijders Blok, Claudia Rozendom, Paul J. Besseling, Jolanda van der Velden, Pim van der Harst, Magdalena Harakalova, Frank G. van Steenbeek

**Affiliations:** 1 Department Clinical Sciences, Faculty of Veterinary Medicine, Utrecht University, Utrecht, the Netherlands; 2 Regenerative Medicine Center Utrecht (RMCU), UMC Utrecht, University of Utrecht, Utrecht, the Netherlands; 3 Department of Cardiology, Division Heart & Lungs, UMC Utrecht, University of Utrecht, Utrecht, the Netherlands; 4 Department Biomolecular Health Sciences, Faculty of Veterinary Medicine, Utrecht University, Utrecht, the Netherlands; 5 Department of Biochemistry, CARIM, Maastricht University, Maastricht, the Netherlands; 6 Netherlands Heart Institute, Holland Heart House, Utrecht, the Netherlands; 7 Department of Nephrology and Hypertension, UMC Utrecht, University of Utrecht, CX Utrecht 3584, the Netherlands; 8 Department of Physiology, Amsterdam Cardiovascular Sciences, Amsterdam University Medical Center, Amsterdam, the Netherlands

**Keywords:** arterial thromboembolism, digital pathology, cardiac fibrosis, cardiac remodeling, nucleus segmentation

## Abstract

Cats frequently develop myocardial remodeling, for example, hypertrophic cardiomyopathy, affecting 14.7% of domestic cats compared to 0.2% of humans, with shared genetic features making them relevant to human disease. Yet features distinguishing clinical outcomes such as heart failure and arterial thromboembolism remain poorly characterized. Using artificial-intelligence-based digital pathology and Oxford Nanopore sequencing, we analyzed myocardial tissue from 37 cats grouped by outcome: arterial thromboembolism, congestive heart failure, or no documented cardiac disease. Myocardial fibrosis was significantly higher in cats with arterial thromboembolism, indicating a distinct fibrotic phenotype. Cats with heart failure showed nuclear hypertrophy, while cats with arterial thromboembolism had increased numbers of small, hematoxylin-dense non-myocyte nuclei. Higher fibrosis was associated with downregulation of mitochondrial and cardiac conduction genes, and nuclear size correlated with proteostasis and stress-response pathways. This multimodal framework reveals distinct histological and molecular profiles by outcome, with relevance for translational hypertrophic cardiomyopathy research.

## Highlights

• AI-based digital pathology quantifies myocardial remodeling in feline cardiac disease
• Cats with arterial thromboembolism show increased myocardial fibrosis and vascular changes
• Distinct nuclear profiles characterize heart failure and thromboembolism outcomes
• Nanopore RNA sequencing identifies gene pathways linked to myocardial tissue phenotypes

## Graphical abstract

**Figure:**
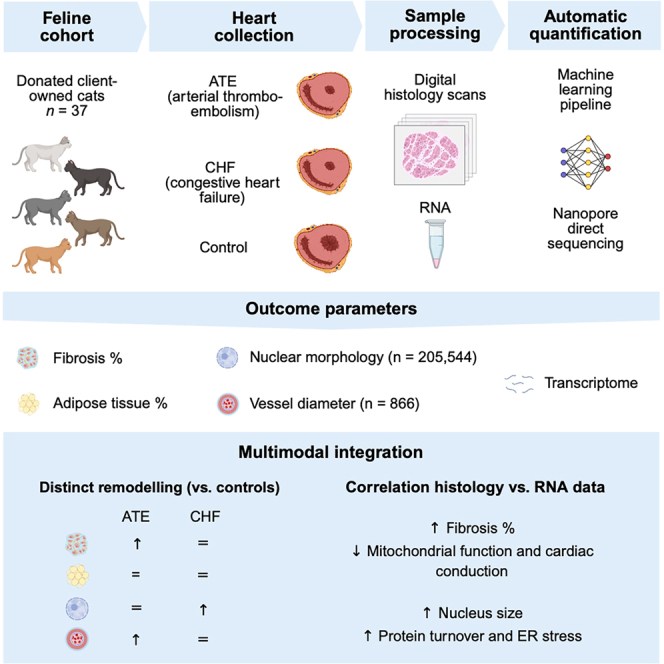

## Teaser

Cardiovascular medicine; Veterinary medicine; Feline cardiology

## Introduction

Hypertrophic cardiomyopathy (HCM) is the most common genetic cardiac disease, affecting at least 0.2% of the general human population.^1^ Despite decades of research, a fundamental clinical question remains unanswered: why do some individuals remain stable for years, while others develop severe complications such as congestive heart failure (HF), arterial thromboembolism (ATE), or sudden cardiac death^2^? Understanding the factors that drive this variability is essential for improving risk stratification and treatment.

Domestic cats provide a naturally occurring model of HCM that closely resembles the human condition in terms of cardiac morphology, genetic predisposition, and clinical complications.^3^^,^^4^ HCM affects around 14.7% of cats and arises spontaneously, making feline patients a valuable, but underused, resource for translational research.^5^ Long-term inbreeding and selective breeding for specific phenotypic traits have turned domestic animals into a magnifying glass for genetic disorders.^6^ Compared to mice, cats offer a more physiologically relevant system,^7^ while still providing a less complex genetic background that can facilitate the identification of disease-modifying mechanisms.

Histopathologically, human HCM is characterized by myocyte hypertrophy, disarray, and variable degrees of fibrosis.^8^ In feline HCM, interstitial remodeling is more consistently documented than myocyte disarray, and morphometric evidence for cardiomyocyte hypertrophy at the cellular level remains inconsistent despite the characteristic macroscopic left ventricular hypertrophy.^9^^,^^10^^,^^11^^,^^12^

Studies involving human pathogenic variant carriers have demonstrated that fibrotic changes can appear even before overt hypertrophy is detected, suggesting that fibrosis may precede subsequent structural remodeling.^13^ However, much of the understanding of myocardial fibrosis in HCM has come from cardiac MRI, surgical biopsies, and tissue from end-stage transplant procedures, all of which have inherent limitations. In cats, interstitial remodeling has been quantified across several studies using different stainings and quantification approaches.^10^^,^^11^^,^^12^^,^^14^ Reported values vary considerably across studies and show substantial within-group heterogeneity, suggesting that fibrosis burden differs among individual cats. Our study extends this work by applying an artificial intelligence (AI)-driven pixel classification approach to complete midventricular transversal sections including the left ventricle, right ventricle, and interventricular septum simultaneously, enabling quantitative fibrosis assessment and evaluation of associations with clinical findings.

ATE is a well-known complication of HCM, both in cats and in humans. In cats, it typically presents as an acute saddle thrombus causing hindlimb paralysis,^15^ whereas in humans it manifests more commonly as stroke or systemic embolism.^16^ Although atrial fibrillation is an established risk factor in human HCM, embolic events also occur in patients without documented atrial fibrillation.^17^ The pathophysiological differences that underlie why some individuals develop thromboembolism and others progress to congestive HF remain largely unexplored.

In human HCM, histopathological assessment of myocardial remodeling is largely restricted to septal myectomy biopsies and explanted hearts, limiting insight into earlier disease stages and regional variation. Feline studies have contributed important pathological characterization of cardiac remodeling,^10^^,^^11^^,^^12^^,^^14^^,^^18^ but translational comparison between species would benefit from quantitative, high-throughput approaches applicable across both. Therefore, we applied a semi-automated digital pathology pipeline to quantify myocardial fibrosis in histological sections from cats with naturally occurring HF, alongside AI-assisted morphometric analysis of nuclear shape, vascular wall-to-lumen ratio, and bulk transcriptomic profiling. We found that fibrosis burden differed markedly between cats that died of ATE versus congestive HF, suggesting that these clinical outcomes are associated with histologically distinct tissue profiles. This demonstrates the feasibility of combining scalable image analysis and transcriptomics to extract meaningful biological associations from naturally occurring cardiac disease, with potential translational relevance for human cardiomyopathy research.

## Results

### Cohort description

Hearts from 37 cats submitted to the Utrecht University Faculty of Veterinary Medicine were collected (Table 1). Most were euthanized due to poor prognosis. Cats were grouped as follows: ATE (*n* = 10), based on hindlimb paralysis, absent femoral pulses, hypothermia, and pallor; congestive HF with cardiomegaly confirmed at necropsy (*n* = 9); and non-cardiac controls (*n* = 18). HF diagnosis was based on clinical signs and accompanied by ultrasound (5/9), echocardiography (1/9), radiographs (1/9), or, in the remaining cases (2/9), by gross pathology findings at necropsy. The average age of cats with a known cardiac death and HF or ATE was 8.63 and 8.40, respectively, while the average age of cats without known cardiac death was 11.77 years.

**Table 1:** Characteristics of the feline cohort

Cat ID	Breed	Age (years)	Sex	Body condition	Reason for euthanasia	Known cardiac death	ATE	Fibrosis %	Myocardial fibrosis %	Adipocyte %
1	DSH	ND	ND	ND	hyperthyroidism	no	no	2.78	1.57	4.22
2	DSH	16	M/N	overweight	acute central neurological symptoms	no	no	3.78	2.79	3.76
3	DSH	10	M/N	underweight	ATE suspicion	yes	yes	12.9	7.99	7.97
4	DSH	11	M/N	optimal weight	lethargy, decreased appetite, mild pancytopenia, FIV positive	no	no	6.67	4.88	11.59
5	DSH	10	M/N	obese	hyperkalemia, urethral obstruction	no	no	3.95	2.76	6.57
6	DSH	23	F/N	underweight	myiasis	no	no	6.26	3.16	5.93
7	DSH	16	M/N	optimal weight	ATE suspicion	yes	yes	7.38	4.85	3.64
9	DSH	17	M/N	ND	unknown	no	no	6.35	3.22	–
10	DSH	8	M/N	ND	heart failure, ATE	yes	yes	13.5	6.6	5.88
11	Birman	4	M/N	underweight	heart failure and mechanical ileus	yes	no	10.26	6.76	3.94
12	DSH	17	M/N	ND	found dead	no	no	5.81	3.03	4.15
13	DSH	5	F/N	optimal weight	HCM	yes	no	5.04	3.62	2.98
14	DSH	10	M/N	obese	heart failure, ATE	yes	yes	8.44	6.57	4.66
15	Maine Coon	14	M/N	ND	found dead, chylothorax and cardiomegaly	yes	no	14.01	11.16	7.97
16	DSH	13	F/N	optimal weight	heart murmur, lameness	no	no	11.44	4.73	9
17	DSH	14	F/N	ND	dyspnea, died in car to clinic. Heart murmur history	no	no	6.28	4.41	2.88
18	Birman	ND	F/N	underweight	sublingual mass	no	no	10.32	5.26	3.87
19	DSH	9	F/N	ND	epilepsia	no	no	5.19	3.69	4.43
20	British Shorthair	2	F/N	optimal weight	dyspnea, enlarged left atrium	yes	no	5.57	4.05	2.84
21	DSH	13	F/N	obese	dyspnea, severe cardiomegaly	yes	no	2.59	1.35	2.34
22	Maine Coon	7	M/N	optimal weight	ATE	yes	yes	9.63	7.03	7.08
23	DSH	13	F/N	ND	ATE, cardiomegaly	yes	yes	22.05	11.58	5.78
24	DSH	15	F/N	underweight	anorexia, epileptic seizure. Thyroid mass removed 1 year before	no	no	6.03	2.95	2.11
25	DSH	12	F/N	ND	anorexia, vomiting	no	no	6.82	4.17	8.2
26	Maine Coon mix	3	M/N	overweight	ATE and dyspnea	yes	yes	–	6.65 (LV only)	2.97
27	DSH	10	F/N	underweight	renal failure	no	no	9.98	6.5	3.03
28	DSH	7	M/N	optimal weight	ATE	yes	yes	10.92	8.62	3.05
29	British Shorthair	0.6	M/I	optimal weight	ibuprofen intoxication	no	no	8.81	5.37	3.54
30	DSH	18	F/N	underweight	dyspnea	yes	no	5.43	3.06	5.02
31	DSH	3	F/N	optimal weight	dyspnea, pericardial effusion	yes	no	–	–	8.66
32	DSH	5	M/N	optimal weight	ATE	yes	yes	14.76	7.37	7.21
33	British Shorthair	7	M/N	overweight	anemia	no	no	12.46	7.93	6.31
34	DSH	ND	ND	unknown	unknown	no	no	10.29	7.07	3.34
35	Maine Coon	ND	ND	unknown	dyspnea, cardiomegaly	yes	no	6.16	2.42	2.32
36	DSH	2	F/N	overweight	renal failure	no	no	7.3	4.66	12.01
37	DSH	10	M/N	optimal weight	congestive heart failure	yes	no	–	–	4.03
38	DSH	5	M/N	optimal weight	ATE	yes	yes	6.19	4.35	4.8

demographic, clinical, and histological data of 37 cats, including breed, age, sex, body condition, cause of death, cardiac status, fibrosis percentage, myocardial fibrosis percentage, and adipocyte percentage. Cause of death refers to the reason for euthanasia unless stated otherwise. Abbreviations: DSH, domestic short hair; N/D, no data; M/N, male neutered; F/N, female neutered; M/I, male intact; ATE, arterial thromboembolism.

### Myocardial fibrosis is elevated in cats with ATE

**Figure 1:**
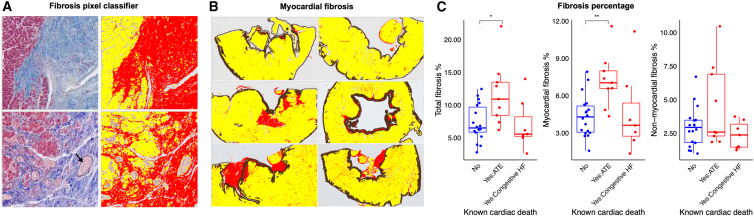
Fibrosis quantification (A) Fibrosis pixel classifier. Representative histological images of fibrotic myocardial tissue stained with Masson’s trichrome (left: blue = fibrosis, red = cardiomyocytes). The corresponding pixel classification outputs (right) display fibrosis (red), cardiomyocytes (yellow), and background. Blood vessel lumens and erythrocytes are classified as background (arrow). (B) Examples of fibrosis quantification (red) in myocardial tissue (yellow) sections. In some samples, excessive endocardial fibrosis was observed. For the quantification of myocardial fibrosis, fibrotic areas at the inner and outer borders were filtered out. The brown line delineates the segmentation borders used to define myocardial fibrosis. (C) Boxplots display mean total fibrosis percentage, mean myocardial fibrosis percentage, and mean non-myocardial fibrosis percentage per outcome group (congestive heart failure [HF], HF with arterial thromboembolism [ATE], and no reported cardiac cause of death), with each dot representing an individual cat (HF: *n* = 7, ATE: *n* = 9, no cardiac death: *n* = 18). Pairwise comparisons were performed using unpaired *t* tests (mean total fibrosis percentage) or Wilcoxon rank-sum tests (mean myocardial and non-myocardial fibrosis percentage), with *p* values adjusted for multiple comparisons using the Benjamini-Hochberg method. Cats with ATE had significantly higher mean total fibrosis percentage and mean myocardial fibrosis percentage compared to cats without a reported cardiac cause of death. No other significant pairwise differences were observed. ∗*p* < 0.05, ∗∗*p* < 0.01.

Fibrosis was quantified using a machine-learning-based pixel classification approach applied to Masson’s trichrome-stained histological sections (Figure 1A). This pixel classification model accurately distinguished blue-stained fibrotic tissue across images with varying staining intensities and filtered out artifacts such as tissue tears and erythrocytes, enhancing the reliability of the quantification. Model performance was evaluated by comparing its predictions to human observer scores across six areas of interest (Figure S1A). Within the cohort, the percentage of fibrosis relative to the total area of cardiomyocytes and fibrosis ranged from 2.59% to 22.05% (Figure 1C). Cats without known cardiac death exhibited a significantly lower fibrosis percentage compared to those with ATE (median 6.51% vs. 10.92%; unpaired *t* test; p_adj_ = 1.52 × 10^−2^). When combining cats with ATE and congestive HF in a “known cardiac death” group, no significant difference was observed between cats with and without a known cardiac death. In these groups, the percentage of collagen deposition ranged from 2.78% to 12.46% and 2.59%–14.01%, respectively. We observed different patterns of distribution of fibrosis among the heart slices, as some hearts showed extensive fibrotic remodeling of the papillary muscles and tendon insertion sites (Figure 1B). To assess myocardial fibrosis specifically, we excluded large subepicardial and subendocardial fibrotic areas connected to the papillary muscles while retaining scar-like fibrosis infiltrating the myocardium. The percentage of these large subepicardial and subendocardial fibrotic areas did not differ significantly between the outcome groups (Figures 1C; Table S3). When focusing solely on myocardial fibrosis, the contrast between cats with ATE and those without a known cardiac death became more pronounced (median 7.03% vs. 4.29%; unpaired *t* test; p_adj_ = 4.62 × 10^−3^), while comparisons involving the congestive HF group remained non-significant.

Supplemental adipocyte quantification (Data S1) showed that adipocytes were predominantly epicardial, with higher surface percentages in the right ventricle than elsewhere in the heart, but no significant differences between outcome groups were found (Figure S2; Table S3). Adipocyte size measurements, available for 29 cats after quality filtering, likewise showed no significant group differences.

### Cats with ATE show a higher vascular wall-to-lumen ratio in large vessels

**Figure 2:**
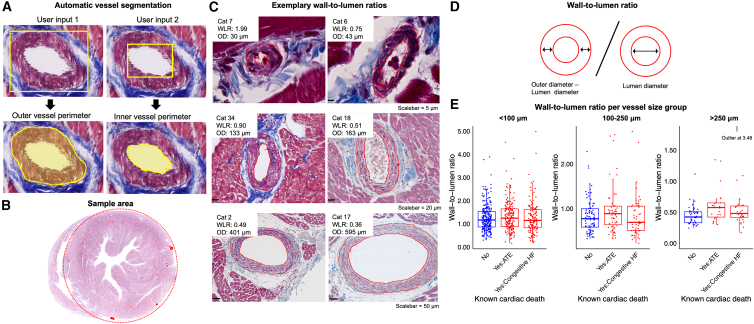
Vessel segmentation (A) The user provides an initial bounding box as input, after which the segment anything model (SAM) automatically segments the vessel structures. (B) Sample area selection. Vessels were segmented in the left ventricle and septum as the region of interest for analysis (dotted red outline). (C) Exemplary wall-to-lumen ratios (WLRs). Histological images of segmented vessels illustrate variations in wall thickness and lumen size across different cats. Calculated WLR and outer diameters (OD) are shown for each example. (D) WLR calculation. Schematic representation of the formula: WLR = (OD − lumen diameter)/lumen diameter. (E) Boxplots show WLR measurements categorized by vessel size group (small: <100 μm, *n* = 573 vessels; medium: 100–250 μm, *n* = 198 vessels; large: >250 μm, *n* = 95 vessels from 35 cats), from 37 cats total, stratified by outcome group (congestive heart failure [HF], HF with arterial thromboembolism [ATE], and no reported cardiac cause of death). Each dot represents one vessel. Overall group differences were assessed using Kruskal-Wallis tests; pairwise comparisons for the large vessel group were performed using a log-Gaussian linear mixed model with cat as a random effect, with *p* values adjusted using the Benjamini-Hochberg method. A nominally significant overall difference in WLR was observed in the large vessel group (Kruskal-Wallis, *p* = 0.016), but no pairwise comparisons reached significance after correction. No significant differences were found in small or medium vessel categories. One outlier in the large vessel group (WLR = 3.48) fell outside the plotted axis range and is indicated.

We used the segment anything model (SAM) to automate vessel segmentation, replacing manual tracing with a more consistent and scalable method (Figure 2A). Arteries and arterioles located in the left ventricle and septum were selected for analysis (Figure 2B). Using this segmentation method, 866 vessels across 37 cats were identified. The number of vessels detected per cat ranged from 11 to 42, with an average of 23.41 ± 7.90 vessels per cat. From the resulting segmentation masks, key vessel parameters were calculated, including inner and outer diameters, wall thickness, wall cross-sectional area, and wall-to-lumen ratio. In large vessels (outer diameter >250 μm), a significant difference in wall-to-lumen ratio among outcome groups was found [H(2) = 8.34; Kruskal-Wallis; *p* = 1.55 × 10^−2^; *n* = 95]. Pairwise comparisons, using a Gaussian linear mixed model (LMM) to account for the unequal number of vessels measured per cat, showed that wall-to-lumen ratio was significantly higher in cats with ATE compared to cats without known cardiac death (median 0.575 vs. 0.423; Gaussian LMM; *p* = 2.56 × 10^−2^; Figure 2E), however, significance was lost after adjustment for multiple comparisons (p_adj_ = 7.67 × 10^−2^). Other vessel parameters did not differ significantly between groups (Table S3).

### Nuclear size and non-myocyte density differ by cardiac outcome

**Figure 3:**
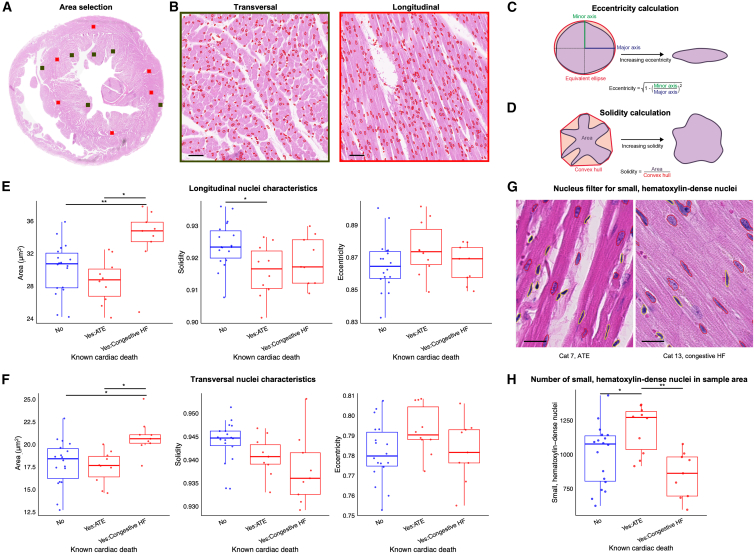
Nucleus segmentation and morphometric analysis (A) Area selection. Six longitudinal and six transversal areas from HE-stained sections were sampled from the left ventricle and septum for nucleus segmentation and analysis. (B) Representative images of nuclei detected in transversal (left, dark gray border) and longitudinal (right, red border) myocardial sections, highlighting differences in nuclear morphology depending on orientation. Scale bars: 50 μm. (C) Eccentricity calculation: defined as the ratio between the minor and major axes of an ellipse fitted to the nucleus, measuring deviation from circularity. (D) Solidity calculation: defined as the ratio of nucleus area to its convex hull area, indicating the degree of nuclear irregularity. (E and F) Nucleus characteristics grouped by outcome group (congestive heart failure (HF), HF with arterial thromboembolism (ATE), and no reported cardiac cause of death), showing area (μm^2^), solidity, and eccentricity in longitudinally cut (E) and transversally cut (F) nuclei. Each dot represents one cat. Pairwise comparisons were performed using estimated marginal means from log-linear mixed models with cat as a random effect, with *p* values adjusted using the Benjamini-Hochberg method. ∗*p* < 0.05, ∗∗*p* < 0.01. (G) Examples of the filtering step used to identify small, hematoxylin-dense nuclei (yellow border) within the quantified area. Nuclei excluded by this filter are indicated by a red border. Cat 7 (diagnosed with ATE) contained 1,365 small hematoxylin-dense nuclei within the quantified area, compared with 595 in Cat 13 (diagnosed with congestive HF). Scale bars: 20 μm. (H) Number of small, hematoxylin-dense nuclei per cat within the sampled area, grouped by outcome group. Pairwise comparisons were performed using unpaired *t* tests with Benjamini-Hochberg correction. Cats with ATE had significantly more small, hematoxylin-dense nuclei than cats with HF and cats without a reported cardiac cause of death. ∗*p* < 0.05, ∗∗*p* < 0.01. *n* = 18 (no cardiac death), 10 (ATE), and 9 (congestive HF) cats.

Nuclei were segmented using CellPose on H&E-stained scanned slides (Figure 3A). For each fiber orientation within the transversal section, six sample areas of 0.25 mm^2^ were analyzed (Figures 3A and 3B). This yielded a total of 111,687 nuclei in transversal cut orientation and 93,857 in longitudinal cut orientation, with an average of 5,555 ± 1,022 nuclei per cat.

Nucleus size differed significantly between outcome groups, with cats that died from congestive HF having the largest nuclei on average (Figures 3E and 3F). In the longitudinal direction, the log LMM model showed a significant effect (*p* = 7.95 × 10^−3^). Post-hoc analysis confirmed that nuclei were larger in cats with congestive HF compared to cats without known cardiac death (median 32.2 μm^2^ vs. 28.2 μm^2^; *p* = 1.95 × 10^−2^, p_adj_ = 2.93 × 10^−2^) and cats with ATE (median 32.2 μm^2^ vs. 26.3 μm^2^; *p* = 3.15 × 10^−3^, p_adj_ = 9.46 × 10^−3^). A similar trend was observed in the transversal direction, where the log LMM model again showed a significant effect (*p* = 1.19 × 10^−2^). Nuclei were significantly larger in cats with congestive HF than in cats without known cardiac death (median 18.7 μm^2^ vs. 16.5 μm^2^; *p* = 1.08 × 10^−2^, p_adj_ = 1.61 × 10^−2^) and cats with ATE (median 18.7 μm^2^ vs. 15.7 μm^2^; *p* = 9.16 × 10^−3^, p_adj_ = 1.61 × 10^−2^). While these results point toward nuclear hypertrophy in the congestive HF group, the size distributions likely reflect a mixture of cardiomyocyte and non-cardiomyocyte nuclei. As a result, differences in the proportion of smaller nuclei between groups, particularly in cats with ATE, may partially mask underlying hypertrophy.

To explore this possibility, we selectively filtered for small, hematoxylin-dense nuclei (<35 μm^2^, upper 0.5 quantile hematoxylin median intensity) consistent with non-myocyte morphology (Figure 3G). Within the same standardized sampling area (6 × 0.25 mm^2^ in the longitudinal plane), significantly more small, hematoxylin-dense nuclei were detected in cats with ATE compared to those with congestive HF (median 1272 vs. 862; *p* = 4.83 × 10^−4^, p_adj_ = 1.45 × 10^−3^) and compared to cats without a known cardiac death (median 1272 vs. 1077; *p* = 2.68 × 10^−2^, p_adj_ = 4.02 × 10^−2^) (Figure 3H). These findings suggest a greater number of non-myocyte nuclei in the ATE group and support the interpretation that elevated numbers of small nuclei may influence overall nuclear size metrics.

Nuclear solidity, a measure of how tightly the nucleus membrane encloses its area, was significantly affected by disease status in both transversal (Log LMM, *p* = 2.83 × 10^−2^) and longitudinal (Log LMM, *p* = 2.55 × 10^−2^) cut directions (Figures 3E and 3F). Increased folding of the nucleus membrane was observed in the nuclei of cats with known cardiac death versus the cats without a known cardiac death, indicated by a trend toward reduced solidity in longitudinally cut nuclei in cats with ATE (median 0.935 vs. 0.927; *p* = 1.60 × 10^−2^, p_adj_ = 4.79 × 10^−2^) and in transversal for cats with congestive HF (median 0.953 vs. 0.949; *p* = 1.73 × 10^−2^, p_adj_ = 5.18 × 10^−2^).

### Myocardial fibrosis correlates with smaller average nuclear size and increased non-myocyte nuclei

**Figure 4:**
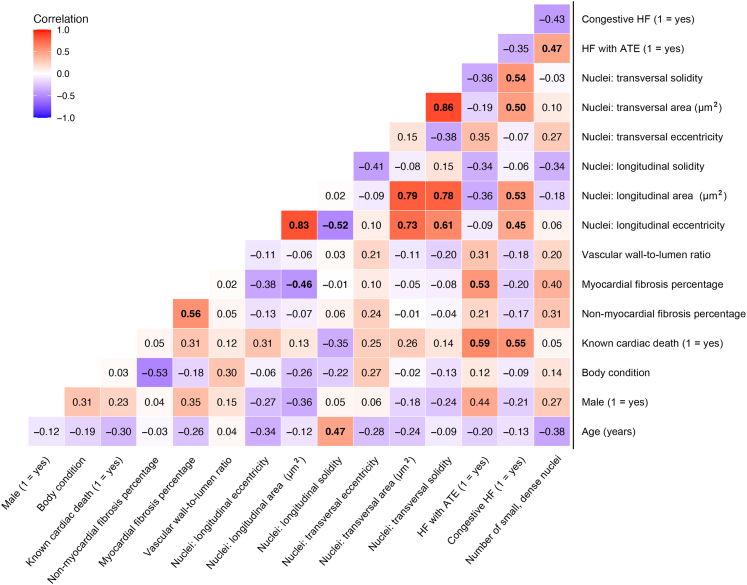
Correlation matrix of phenotype quantifications This heatmap displays pairwise correlation coefficients between histological measurements and individual characteristics from up to 37 cats, where not all measurements were available for all individuals (see Table 1). Pearson or Spearman correlation was used depending on the normality of each variable (see Table S4). Positive correlations are shown in red and negative correlations in blue, with color intensity reflecting correlation strength. Numbers indicate correlation coefficients. Correlations with *p* < 0.05 are indicated in bold.

A correlation analysis identified several significant relationships among the studied phenotypic traits (Figure 4). Non-myocardial fibrosis percentage showed a strong positive correlation with myocardial fibrosis percentage (*r* = 0.56, Spearman; *p*_adj_ = 7.97 × 10^−3^), confirming that these two types of fibrosis tend to co-occur. A particularly relevant finding was the negative correlation between myocardial fibrosis percentage and nucleus size in longitudinal sections (*r* = −0.46, Pearson; *p*_adj_ = 4.47 × 10^−2^) but not in the transversal sections (*r* = −0.05, Pearson; *p*_adj_ = 8.81 × 10^−1^). This suggests that increased fibrosis is associated with the presence of smaller nuclei that can only be observed in longitudinal cuts. This is further supported by the positive correlation between the number of small, hematoxylin-dense nuclei and myocardial fibrosis percentage (*r* = 0.40, Pearson; *p*_adj_ = 9.81 × 10^−2^).

### Transcriptomic profiling links tissue phenotypes to distinct pathway signatures

**Figure 5:**
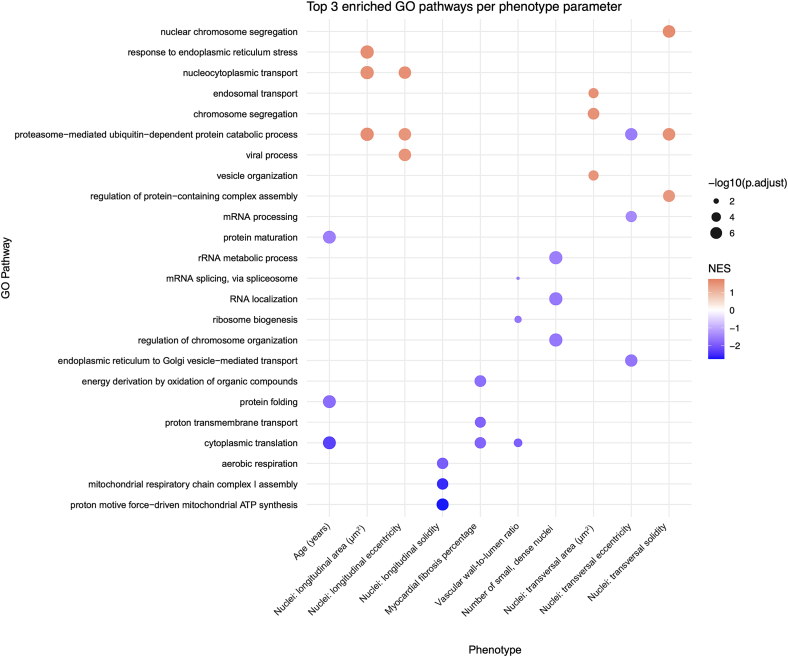
Transcriptome-wide enrichment of biological pathways associated with histological and nuclear phenotype parameters Dot plot showing the top three significantly enriched Gene Ontology Biological Process terms per phenotype parameter (adjusted *p* < 0.05, Benjamini-Hochberg correction) across 12 cats with available transcriptomic data. Dot color represents the normalized enrichment score (NES), indicating the direction and strength of the association; dot size reflects -log_10_ of the adjusted *p* value. Parameters include age, nuclear morphology (area, eccentricity, and solidity) measured in longitudinal and transversal myocardial sections, myocardial fibrosis percentage, vascular wall-to-lumen ratio, and number of small, hematoxylin-dense nuclei. See also Table S5.

Oxford Nanopore long-read sequencing data were available for 12 cats (cats 10, 11, 15, 16, 23, 25, 30, 31, 35, 36, 37, and 38). A total of 28,618,267 reads were generated through Nanopore sequencing, of which 92% (26,478,932 reads) passed the Q-score threshold of 7. The mean sequencing depth was 35× per gene, enabling robust transcriptome-wide expression analysis. Genes were ranked by Spearman correlation with histological parameters, and pathway enrichment was assessed using normalized enrichment scores (NES). The top three pathways per trait are shown in Figure 5 and the top 10 in Table S5.

Higher fibrosis percentage was linked to reduced expression of genes involved in cardiac conduction (NES = –2.04, p_adj_ = 4.37 × 10^−4^), mitochondrial organization (NES = –1.61, p_adj_ = 4.46 × 10^−4^), and cardiac muscle cell action potential (NES = –1.96, p_adj_ = 1.68 × 10^−3^). These included ion-handling and membrane-related genes such as *RYR2*, *PLN*, *CACNA1C*, *SCN3A*, and *DSP*.

Nucleus size was positively associated with stress and proteostasis pathways in both cut directions. These pathways included proteasome-mediated ubiquitin-dependent protein catabolic process (NES = 1.71 and 1.52, p_adj_ = 2.59 × 10^−8^ and 1.98 × 10^−4^) and response to endoplasmic reticulum stress (NES = 1.70 and 1.60, p_adj_ = 2.59 × 10^−8^ and 2.94 × 10^−4^). Key genes in these pathways, such as *KLHL30*, *LRRK2*, *FAF1*, and *UFD1*, contribute to ubiquitin ligase activity, intracellular transport, degradation of misfolded proteins, and cellular stress responses. These findings suggest that larger nuclei may reflect activation of protein quality control systems and stress adaptation.

Nuclear eccentricity showed direction-specific associations. In longitudinal sections, higher nuclear eccentricity, reflecting more elongated nuclei, was positively associated with nucleocytoplasmic transport (NES = 1.69, p_adj_ = 1.90 × 10^−7^). In transversal sections, by contrast, higher eccentricity was associated with reduced activity of nucleocytoplasmic transport (NES = –1.59, p_adj_ = 1.32 × 10^−5^), as were pathways related to chromosome organization (NES = –1.81, p_adj_ = 2.85 × 10^−7^) and mitotic cell-cycle regulation (NES = –1.47, p_adj_ = 2.59 × 10^−5^), including the genes *TGFB1*, *BUB1*, and *CDK2*. Because nuclear shape is influenced by both cell type and mechanical interplay, variation in eccentricity likely reflects a combination of cardiomyocyte remodeling and differences in local cell population, including contributions from fibroblasts and other non-myocyte cells.

Higher nuclear solidity (reflecting less membrane folding) in longitudinal cuts was linked to reduced expression of genes related to mitochondrial energy production, including proton-motive-force-driven mitochondrial ATP synthesis (NES = −2.74, p_adj_ = 5.10 × 10^−7^) and aerobic respiration (NES = −2.12, p_adj_ = 2.66 × 10^−6^).

The number of small, hematoxylin-dense nuclei was negatively associated with RNA processing and translation-related pathways, including RNA localization (NES = −1.78, p_adj_ = 3.24 × 10^−8^) and regulation of translation (NES = −1.67, p_adj_ = 3.24 × 10^−8^). These associations may reflect shifts in cellular composition in samples with increased number of small, hematoxylin-dense nuclei, where non-myocyte populations typically exhibit lower baseline transcriptional activity compared to cardiomyocytes.

Vascular wall-to-lumen ratio showed negative enrichment for ribosomal and translational pathways. These included cytoplasmic translation (NES = –2.08, p_adj_ = 4.04 × 10^−4^), ribosome biogenesis (NES = –1.77, p_adj_ = 2.18 × 10^−3^), and rRNA metabolic process (NES = –1.70, p_adj_ = 1.74 × 10^−2^).

Finally, age was negatively associated with cytoplasmic translation (NES = –2.47, p_adj_ = 7.11 × 10^−8^), protein folding (NES = –1.92, p_adj_ = 7.11 × 10^−8^), and nucleocytoplasmic transport (NES = –1.66, p_adj_ = 7.55 × 10^−6^), suggesting age-related downregulation of proteostasis and RNA handling pathways in myocardial tissue.

Supplemental qPCR analysis of left ventricular tissue from 21 cats (Data S1) confirmed a *MYH6*/*MYH7* ratio resembling that of human rather than murine myocardium, with significantly higher *MYH7* expression (Figure S3A). We also confirmed that *NPPA* expression was significantly higher in cats with a known cardiac death, with no difference between ATE and congestive HF subtypes (Figure S3B; Table S3).

### Fibrosis, vascular remodeling, and aging share reduced translational activity

To examine whether histologically correlated phenotypes also share transcriptomic signatures, we compared pathway overlap across the top 10 enriched gene sets per phenotype (Table S5). Nucleus shape and size phenotypes showed broad overlap in pathways related to RNA processing and protein homeostasis. Notably, longitudinal and transversal solidity, despite only a weak histological correlation (r = 0.15), both showed strong downregulation of proton-motive-force-driven mitochondrial ATP synthesis (NES = −2.74 and −2.52, respectively). Myocardial fibrosis, wall-to-lumen ratio, and age showed limited histologic correlation with one another, but all shared cytoplasmic translation among their top downregulated pathways, pointing to a common reduction in translational activity across these traits. Age and the number of small, hematoxylin-dense nuclei were inversely correlated histologically (r = −0.38) yet shared three downregulated pathways (RNA localization, mRNA processing, and proteasome-mediated ubiquitin-dependent protein catabolic process).

## Discussion

This explorative study applies quantitative histological and transcriptomic analyses to cats grouped by clinical outcome, combining AI-based tissue characterization with molecular profiling in a naturally occurring cohort. We quantified fibrosis and adipocyte percentage, vascular remodeling, nuclear morphology, and RNA expression.

The integration of AI-based quantification significantly enhanced the resolution and scalability of histological analyses. By employing a label-free, machine-learning-driven pipeline for fibrosis, vascular morphology, and nuclear segmentation, we achieved robust, reproducible data without the constraints of antibody-dependent methods. Such an approach is transferable across species, facilitating translational research without species-specific reagents or labels. Additionally, the high-resolution analysis, particularly at the level of nuclear morphology, allowed us to capture subtle yet potentially clinically relevant differences in cellular remodeling.

Cats presenting with ATE showed significantly increased myocardial fibrosis compared to those with congestive HF and controls, suggesting that ATE is associated with a distinct myocardial tissue profile rather than simply representing an advanced disease stage. These findings underscore the complexity and heterogeneity of feline cardiac diseases, highlighting a critical knowledge gap regarding the mechanisms underlying fibrosis-associated arrhythmic and thromboembolic complications. Similar patterns have been observed in human patients, where fibrosis correlates with increased arrhythmic and thromboembolic risk,^16^^,^^19^ yet the specific drivers of this variability remain poorly understood.

Myocardial fibrosis was effectively classified and quantified using a QuPath-based pixel classifier manually trained on a subset of the data, consistent with prior studies.^20^^,^^21^ Fibrosis levels varied widely among feline samples and were generally lower than those reported in human end-stage HCM,^22^ suggesting our cohort captures a broader spectrum of cardiac remodeling. Notably, fibrotic lesions were present even in hearts without diagnosed cardiac disease, possibly reflecting the high prevalence of subclinical HCM in cats.^5^ Transcriptomic profiling revealed that greater myocardial fibrosis was associated with downregulation of genes related to energy production and electrical function. These findings align with human HCM, where fibrosis progression accompanies mitochondrial dysfunction and electrical instability.^23^

The anatomical distribution of fibrosis ranged widely, with prominent patches around the papillary muscles and subendocardial or epicardial regions. To improve specificity, we excluded fibrotic patches associated with anatomical structures like false tendons, which are commonly observed in feline and human hearts but lack clear clinical significance^24^^,^^25^

Cats with congestive HF without ATE showed significant nuclear hypertrophy, which is consistent with a cardiomyocyte response to stress, such as mechanical overload or impaired relaxation.^26^ Transcriptomic analysis linked larger nuclear areas to increased expression of protein turnover and endoplasmic-reticulum-stress-related genes, suggesting a broader adaptive cellular response. Increased nuclear size has been previously associated with enhanced transcriptional activity and chromatin remodeling in cardiomyocytes under mechanical and metabolic stress.^27^ In contrast, cats with ATE exhibited high fibrosis but not increased nuclear size, likely due to a higher proportion of smaller, non-myocyte nuclei. This interpretation was supported by a positive correlation between fibrosis and the number of small, hematoxylin-dense nuclei. In feline HCM, the interstitial compartment contains a heterogeneous non-myocyte population, including increased numbers of Iba1-positive macrophages and interstitial cells of fibroblastic or vascular phenotype, with CD34 expression also identified among interstitial cells.^10^^,^^11^ Given the diversity of non-myocyte cell types reported in feline HCM, immunohistochemical characterization will be needed to establish the cellular composition of this population in relation to clinical outcome.

Myocardial inflammation is an area of ongoing investigation in human HCM, particularly regarding its role in thromboembolic complications.^28^^,^^29^^,^^30^ In feline HCM, remodeling enzyme transcription has been found to be elevated across cases, while inflammatory marker expression was specifically increased in cats with ATE.^31^ Our finding of increased small, hematoxylin-dense nuclei in cats with ATE may reflect a similar expansion of the non-myocyte compartment, though the identity of these cells remains unresolved. The feline model provides an opportunity to further dissect these processes through immunohistochemical and spatial transcriptomic approaches.

Vascular remodeling, another critical aspect of HCM pathology in humans,^32^ showed trends toward increased vessel wall-to-lumen ratios in ATE cats, though differences were not significant after correction. Further exploration in larger cohorts may clarify this association.

Taken together, our findings demonstrate that critical pathological processes, including fibrosis, vascular remodeling, non-myocyte cell presence, and nuclear morphometry, can be integrated and spatially resolved using a high-throughput, label-free image analysis pipeline. This capability allowed us to characterize histological and molecular variation across multiple biological layers. This integrative approach offers valuable mechanistic insights relevant to thromboembolic complications in human cardiology and presents a powerful tool for future studies aiming to unravel complex pathological interactions and identify therapeutic targets in cardiomyopathies. In contrast to human septal myectomy samples and mouse models, which have key limitations, the domestic cat offers a spontaneous, accessible model for studying myocardial remodeling across a range of clinical severities. The availability of whole-heart tissue from naturally affected animals provides opportunities to investigate the timing and effectiveness of potential therapeutic interventions, such as anti-fibrotic or anti-inflammatory approaches.

### Limitations of the study

Our cohort was grouped by clinical outcome and reason of euthanasia rather than by a formal, multi-level HCM diagnosis incorporating clinical, gross pathological, and histopathological criteria. A full ante-mortem cardiological examination was not available in the emergency setting in which tissue was collected. Ante-mortem cardiac imaging was limited, and cardiomegaly was predominantly confirmed at gross pathological examination during necropsy, without standardized echocardiographic measurements or cardiac-to-bodyweight ratios. While HCM is the most common underlying cause of both ATE and cardiomegaly-associated HF in cats, other cardiac diseases may also be involved. Consequently, our cohort may cover a range of cardiac conditions rather than a single disease entity at different stages. Non-cardiac causes of congestive HF, such as hyperthyroidism or systemic hypertension, were not systematically excluded. The grouping by clinical endpoint (HF with or without ATE) emerged from the data during analysis rather than being imposed *a priori*.

Nuclear morphology was measured across all cell types, making it unclear whether observed changes reflect cardiomyocyte remodeling or increased fibroblasts or immune cells. Non-myocyte nuclei were classified by morphology alone, without immunohistochemical confirmation. While transcriptomic data revealed associations with morphological traits, the cross-sectional design precludes causal inference. Although our cohort of 37 cats is substantial for histology- and transcriptomics-based cardiac research in naturally occurring models, larger datasets would improve statistical power and allow more robust subgroup analyses, particularly for vascular parameters.

## Resource availability

### Lead contact

Requests for further information and resources should be directed to and will be fulfilled by the lead contact, Frank van Steenbeek (f.g.vansteenbeek@uu.nl).

### Materials availability

This study did not generate new unique reagents.

### Data and code availability

• Transcriptome data have been deposited at NCBI Gene Expression Omnibus repository as GSE296746 and are publicly available as of the date of publication.
• All original code has been deposited at GitHub and is publicly available at https://github.com/CardiOmnics/feline-myocardial-remodeling-2026 as of the date of publication.
• Pixel classifier files (.json) and the custom Cellpose model have been deposited at Zenodo and is publicly available at https://doi.org/10.5281/zenodo.19085472 as of the date of publication. Any additional information required to reanalyze the data reported in this paper are available from the lead contact upon request.

## Acknowledgments

We acknowledge the Utrecht Sequencing Facility (USEQ) for providing sequencing service and data analysis. USEQ is subsidized by the University Medical Center Utrecht and the Netherlands X-omics Initiative (10.13039/501100003246NWO project 184.034.019). We would like to thank Ali Nassar, Karen Rowena Gaar-Humpreys, and Alysia Postma for their practical support in histology and Aryan Vink, Domenico Castigliego, and Erica Siera-de Koning (10.13039/100020950Department of Pathology, 10.13039/501100003761UMCU Utrecht) for assistance in obtaining full slide scans.

This work was supported by the 10.13039/100018890Dutch Cardiovascular Alliance (10.13039/100018890DCVA) grant (DOUBLE-DOSE no. 2020B005).

## Author contributions

Conceptualization, F.G.v.S. and M.H.; methodology, T.C.F.S.; investigation, T.C.F.S., A.H.H., B.J., C.J.B.S.B., and C.R.; writing – original draft, T.C.F.S. and A.H.H.; writing – review and editing, A.H.H., G.C.M.G., C.N.v.d.W., R.J.A.V., J.v.d.V., P.v.d.H., F.G.v.S., and M.H.; formal analysis, T.C.F.S. and B.J.; resources, A.H.H. and P.J.B.; supervision, F.G.v.S. and M.H.

## Declaration of interests

The authors declare no competing interests.

## Declaration of generative AI and AI-assisted technologies in the writing process

During the preparation of this work, the authors used Claude (Anthropic) and ChatGPT (OpenAI) in order to improve clarity, conciseness, and readability of the text. After using this tool or service, the authors reviewed and edited the content as needed and take full responsibility for the content of the publication.

## STAR★Methods

### Key resources table

**Table:** 

REAGENT or RESOURCE	SOURCE	IDENTIFIER
**Biological samples**		

Feline myocardial tissue, formalin-fixed and paraffin-embedded (midventricular transversal slices)	Postmortem donation from client-owned cats, Utrecht University	N/A
Feline left ventricular free wall tissue for RNA isolation	Postmortem donation from client-owned cats, Utrecht University	N/A

**Chemicals, peptides, and recombinant proteins**		

Standard histology reagents (4% buffered formaldehyde, ethanol, paraffin, hematoxylin and eosin, and Masson’s trichrome components: Celestine Blue, Mayer’s hematoxylin, Biebrich Scarlet-Acid Fuchsin, Aniline Blue)	Routine histology service	Various suppliers
iQ SYBR Green Supermix	Bio-Rad	Cat. no. 1708880

**Critical commercial assays**		

RNeasy Mini Kit	Qiagen	Cat. no. 74104
iScript cDNA Synthesis Kit	Bio-Rad	Cat. no. 1708891
Direct RNA Sequencing Kit	Oxford Nanopore Technologies	SQK-RNA004
FLO-MIN106 R9.4.1 Flow Cell	Oxford Nanopore Technologies	FLO-MIN106

**Deposited data**		

Raw Oxford Nanopore direct RNA sequencing reads, *n* = 12 cats	This paper	NCBI Gene Expression Omnibus
Pixel classifier files (.json) and custom Cellpose model	This paper	Zenodo
Analysis code (QuPath Groovy scripts, R analysis scripts)	This paper	GitHub
Felis catus reference genome, assembly Felis_catus_9.0 (felCat9)	Ensembl	GCA_000181335.4

**Experimental models: Organisms/strains**		

Domestic cat (Felis catus), mixed breeds, postmortem	Client-owned, donated for research after decision to euthanize for clinical reasons unrelated to this study	N/A

**Oligonucleotides**		

qPCR primers for NPPA, MYH6, MYH7, DSG2, RPS5, HPRT1, YWHAZ (sequences and annealing temperatures listed in Table S2)	This paper	See Table S2

**Software and algorithms**		

QuPath (digital pathology platform, pixel classifiers, scripting)	Bankhead et al.^33^	v0.5.1; RRID:SCR_018257; https://qupath.github.io
Cellpose (custom-trained nuclei model, two rounds of human-in-the-loop refinement)	Pachitariu and Stringer^34^	v2.0; RRID:SCR_021716
Segment Anything Model (SAM), checkpoint vit_l, applied via the QuPath SAM plugin v0.4.0 [ref.^35^]	Kirillov et al.^36^	https://github.com/facebookresearch/segment-anything
R	R Foundation for Statistical Computing	v4.4.0 (RStudio build 2024.04.1+748)
Full list of R packages used in the analysis pipeline	Various	See GitHub repository
ONT MinKNOW Software	Oxford Nanopore Technologies	v22.10.6
Guppy basecaller	Oxford Nanopore Technologies	v6.3.9

**Other**		

NanoZoomer XR slide scanner, 20×	Hamamatsu Photonics	Instrument
GridION ×5 Mk1 sequencer (device ID GXB03452)	Oxford Nanopore Technologies	Instrument

### Experimental model and study participant details

#### Animal source and ethical oversight

Cardiac tissue was obtained postmortem from client-owned domestic cats whose owners provided informed consent for the donation of the cadaver for research purposes. The decision to euthanize each animal was made by the attending veterinarian together with the owner, based solely on the clinical condition and welfare of the animal, and was entirely independent of this study. No experimental procedures were carried out on live animals. Carcasses were stored at 4 °C for a maximum of 48 h prior to processing. Because no procedures were performed on live animals and euthanasia was decided on welfare grounds unrelated to this work, the study did not fall within the scope of the Dutch Animals Act, and no institutional ethics or Animal Welfare Body review was required.

#### Cohort composition and group assignment

The cohort is described in Table 1, including age, sex, breed, body condition and reason for euthanasia. Animals were grouped based on the cause of death documented at the time of euthanasia: arterial thromboembolism (ATE), congestive heart failure (HF), and a comparator group with no documented cardiac cause of death. Because more than 90% of feline ATE has a cardiogenic origin,^37^ both the ATE and congestive HF groups were considered to have a known cardiac cause of death. A transcriptomic subcohort of 12 cats was selected (cats 10, 11, 15, 16, 23, 25, 30, 31, 35, 36, 37, and 38) to span the range of clinical outcomes and histological fibrosis observed across the full cohort. Sex was recorded for 34 of 37 animals (males: 19, females: 15). The influence of sex on study outcomes was examined where data were available; no significant associations were identified.

### Method details

#### Tissue collection and processing

After euthanasia, midventricular transversal cardiac slices were fixed in 4% buffered formaldehyde, transferred to ethanol, and embedded in paraffin. Hematoxylin and eosin (HE) staining was performed on the Leica Autostainer XL. Masson’s trichrome staining was used to visualize fibrosis in cardiac tissue sections. Nuclei were stained with Celestine Blue followed by Mayer’s Hematoxylin. Cytoplasm and muscle were stained red with Biebrich Scarlet-Acid Fuchsin, and collagen fibers were stained blue using Aniline Blue. Tissue slides were scanned using a Nanozoomer XR (Hamamatsu, Hamamatsu City, Japan) at 20× magnification, from which digital images were captured.

#### Digital pathology workflow

##### Fibrosis quantification

Fibrosis was quantified using Masson’s trichrome-stained sections. Tissue sections were scanned in total, yielding a field of vision containing cardiac tissue and non-tissue background. Variability in staining intensity and color precluded reliable results from thresholding methods. Therefore, a pixel classification approach using QuPath^33^ version 0.5.1 was used to distinguish fibrous tissue from cardiomyocytes and background. A tissue thresholding step was implemented to separate tissue from the background, enhancing computational efficiency. The thresholding parameters were: very low resolution (14.7 μm/pixel), a smoothing sigma of 5, and a channel average threshold of 225 with a Gaussian prefilter. Tissue fragments smaller than 1 × 10^6^ μm^2^ were excluded from analysis. The pixel classifier for fibrosis quantification was trained using the Random Trees algorithm. Training samples were collected from five representative images, where fibrosis, cardiomyocytes, and background areas were manually annotated. Erythrocytes were categorized as background. The model operated at a resolution of 0.91 μm/pixel, utilizing RGB channels and features set to Gaussian and weighted deviation. Fibrosis percentage was calculated as the ratio of fibrosis area to the sum of fibrosis and cardiomyocyte areas. Occasionally, areas such as papillary muscles and tendinous structures contained large regions of connective tissue, potentially skewing the data. Therefore, these areas were excluded through an additional automatic filtering step. Both endocardial connective tissue and epicardial fibrosis were filtered out to separate myocardial fibrosis. This was achieved by running a custom script in QuPath to automatically select a 700 μm region inward from the tissue border. Within these selected regions, a fibrosis classification model operating at a resolution of 3.6 μm/pixel, using RGB channels and Gaussian features, was applied to identify objects larger than 4,000 μm^2^. After the model application, selected areas were manually reviewed and adjusted when necessary. These excluded regions were omitted from subsequent fibrosis quantification following the above-mentioned procedure.

##### Adipocyte area quantification

Percentage of surface area of fat tissue was determined in HE-stained sections. A training set was established by annotating adipocytes, cardiomyocytes, and background in 10 representative images. The Random Trees algorithm was used to train the classifier at a low resolution of 7.25 μm/pixel, employing RGB channels and features including Gaussian, Laplacian of Gaussian, weighted deviation, gradient magnitude, and structure tensor coherence. A subset of slides (cats 10, 15, 23, 25, 30, and 36) were processed in a second staining and scanning batch, which caused noticeable differences in staining intensity. For these samples, seven additional training areas were manually annotated, and a new classification model was trained using identical parameters. Due to the low resolution required to differentiate adipocytes from tissue tears and blood vessels, a separate high-resolution tissue thresholding model (0.91 μm/pixel) was employed to calculate total tissue area for percentage calculations. Percentage of adipose tissue was computed as the ratio of adipocyte area to the sum of adipocyte and cardiomyocyte areas.

##### Adipocyte size analysis

Adipocyte size was analyzed using a modified version of a previously described method by making a pixel classifier for adipocyte membranes.^38^ This analysis focused on identifying individual adipocytes with high certainty. A new classification model, more sensitive than the quantification model but prone to false positives (e.g., tissue tears), was developed to isolate regions with dense adipocyte populations. Objects larger than 10,000 μm^2^ were retained, and holes smaller than 3,000 μm^2^ were filled. To identify individual adipocytes, a high-resolution pixel classifier was developed to detect adipocyte membranes. The classifier operated at a resolution of 0.45 μm/pixel, utilizing Gaussian features exclusively. Detected objects were filtered using a minimum size threshold of 70 μm^2^, and holes smaller than 30 μm^2^ were filled to ensure an accurate representation of adipocyte structures. For each detected object, morphological parameters including size, circularity, solidity, maximum diameter, and minimum diameter were calculated. However, initial detections included non-adipocyte structures, necessitating an additional filtering step. To enhance specificity, we plotted circularity against size for all detected objects. This allowed visual identification of distinct subpopulations, representing likely adipocytes versus artifacts. Thresholds were then manually set based on clear visual separation observed in this plot (illustrated in Figure S2C), selecting only objects consistent with adipocyte morphology. The accuracy of this refined subset was subsequently validated through visual inspection. This filtered dataset was then utilized for adipocyte size measurements.

##### Vessel segmentation

Vessel object segmentation was performed using the Segment Anything Model (SAM) developed by Meta.^36^ SAM utilizes prompt-based techniques, such as bounding boxes or clicks, to generate object masks, relying on deep learning principles and extensive pretraining to generalize across diverse image types. For this analysis, the SAM model vit_l (large) was applied. For integration with QuPath, the SAM plugin v0.4.0 was used to facilitate segmentation workflows.^35^ The plugin enables local execution, thereby enabling sensitive image analysis tasks. The analysis focused on transversely cut arterioles located in the middle third of the left ventricle and septum. To segment these vessels, bounding boxes were manually drawn around their inner and outer boundaries. The SAM-generated masks were then reviewed for accuracy, and erroneous segmentations were removed. Notably, no manual drawing was used for vessel outlines, ensuring consistency and reliance on the SAM-generated segmentation. Only transversally cut vessels that were correctly segmented (lumen and tunica intima + tunica media) were included in the study. For larger vessels with a visible tunica adventitia, only its dense connective tissue portion was segmented, while the surrounding loose connective tissue was excluded. Due to variability in the number of transversally cut arterioles per image, the number of segmented vessels varied across samples. Vessel characteristics were derived from segmented images by calculating diameters based on the vessel areas. The Outer Diameter (OD) and Lumen Diameter (LD) were computed as the equivalent diameters of circles with the same area, using the formula: $2\times \sqrt{area/\pi }$

From these diameters, additional vessel metrics were calculated:

• Wall Thickness (WT): (*OD*-*LD*)/2
• Wall-to-Lumen Ratio (WLR): (*OD*-*LD*)/*LD*
• Wall Cross-Sectional Area (WCSA): *π*/4×(*OD*^2^-*LD*^2^)

##### Nucleus segmentation and small non-myocyte nucleus identification

To segment cardiomyocyte nuclei, we trained a custom Cellpose model within QuPath version 0.5.1, using a pretrained nuclei model as a starting point. Training was conducted in two rounds using a human-in-the-loop approach, refining the model based on manual corrections.^34^ In the first round, nine diverse regions from different HE scanned slides were selected, ensuring a collection of longitudinal and transversal nuclei. Within each region, nuclei in two 10,000 μm^2^ rectangular areas were annotated using a combination of manual labeling and watershed segmentation. This resulted in a training set of 304 nuclei, split equally into training and validation sets.

The second training round incorporated model predictions from nine new areas, where corrections were made to refine accuracy, particularly in dense or variably stained regions. Segmentation results were evaluated using Intersection over Union (IoU), precision, accuracy, F1-score, and Panoptic Quality, with a mean IoU of 0.75 (±0.07 SD) and precision of 0.98 (±0.04 SD), indicating reliable performance (Table S1). The final model was then applied to six longitudinal and six transversal regions (250,000 μm^2^ each) in different areas of the left ventricle and septum. For each detected nucleus, area, perimeter, minimum diameter, and maximum diameter were extracted for downstream analysis.

Additionally, we identified a population of ‘small, hematoxylin-dense nuclei’ in the longitudinal sections as a surrogate for non-myocyte cell infiltration. To account for inter-sample variability in hematoxylin staining intensity, a dynamic threshold was computed per section by selecting the top 50% of nuclei with the highest median hematoxylin signal. Nuclei meeting this criterion were further filtered based on size (<35 μm^2^). These thresholds were based on empirical inspection of nuclei with non-myocyte morphology. The resulting population was visually validated in several cases to confirm the exclusion of larger cardiomyocyte nuclei.

#### Reverse transcription quantitative PCR (RT-qPCR)

Total RNA was isolated from left ventricular free wall tissue using the RNeasy Mini Kit (Qiagen, Venlo, The Netherlands) including on-column DNase treatment. Complementary DNA was synthesized with the iScript cDNA Synthesis Kit (Bio-Rad, Veenendaal, The Netherlands) following the manufacturer’s instructions. Reactions were prepared using SYBR Green Supermix (Bio-Rad) and run on a CFX384 Touch Real-Time PCR Detection System (Bio-Rad). RPS5, HPRT-1, and YWHAZ were used as reference genes for normalization. All primer sequences with their annealing temperatures can be found in Table S2. qPCR was performed with a two-step gradient protocol optimized for each primer set, ensuring specific amplification. No-template controls (NTCs) were included to detect contamination, and a standard dilution series was used to confirm linear amplification efficiency for each primer pair. To validate product identity, qPCR products were sequenced using the ABI 3500 XL Genetic Analyzer (Applied Biosystems, Carlsbad, CA, USA) and aligned to the feline reference genome to ensure the correct genes were amplified. The relative expression levels of NPPA, MYH6, MYH7, and DSG2 were normalized using the reference genes and calculated using the 2^−ΔΔCT^ method.

#### Bulk Oxford Nanopore direct RNA sequencing

Bulk direct RNA sequencing was performed on the subcohort of 12 cats. Total RNA was isolated from left ventricular free wall tissue immediately adjacent to and above the midventricular histological section using the RNeasy Mini Kit (Qiagen) and stored at −80 °C. RNA quality control was performed by the Utrecht Sequencing Facility (USEQ). After the mRNA selection, libraries were prepared using the Direct RNA sequencing kit (SQK-RNA002, Oxford Nanopore), which enables RNA molecules to be sequenced directly and preserve base modifications. Multiplexing was enabled using four barcodes (barcode 1: GGCTTCTTCTTGCTCTTAGG, barcode 2: GTGATTCTCGTCTTTCTGCG, barcode 3: GTACTTTTCTCTTTGCGCGG, barcode 4: GGTCTTCGCTCGGTCTTATT). The adapters were added according to the manufacturer’s instructions. The libraries were sequenced on FLO-MIN106 flow cells, and for the first run, the GridION sequencing device was used with the ONT MinKNOW Software.

Sequencing reads in the fastq format were mapped against the felCat9 genome (Ensembl sourced), and bam files were split per barcode at the USEQ. All HDF5 files from the GridION platform were base-called using Guppy (v6.3.9), the proprietary software distributed by Oxford Nanopore. The samples were sequenced using the GridION X5 Mk1 (GXB03452). The full run duration lasted a total of 72 h, with an estimated number of bases of 688.6 Mb and a total reads generated of 910.87 k for the GridION. Furthermore, the bases were called on a minimum Q score of >7. All data were single-end reads. The reads of the data were added to the online Galaxy data analysis platform history. This was done by uploading the datasets in their respective formats and extracting the reads with the FASTQ-Groomer tool. The reads first required pre-processing, which was done using the fastP tool. The quality of the original reads was checked with fastQC, and subsequently, based on the results, the reads were filtered on a Phred score of >7 in BAM format, which was then used for further analysis.

The obtained files were referenced using Minimap2, a versatile sequence alignment program for a large reference database, due to the presence of Nanopore long reads. Minimap2 was used with the parameters set by using a genome from history and build index, selected with no preset options. All other parameters were left to their default state, as implied by the Galaxy tool. The BAM files obtained from USEQ were compared against the newly mapped files of Minimap2, before being followed up by FeatureCounts, a read summarization program suitable for counting reads. The files were compared with differences of Phred score >7 and Phred score >15, where the differences were annotated to set up a quality report regarding the obtained files. For further analysis, a Phred score of >7 was chosen due to the reference to other analyses.

A total of 25.4 million reads were mapped, accounting for 95% of the produced reads. Enrichment analysis revealed that the average read length was 1,200 bp, with the longest read exceeding 4,800 bp, highlighting the platform’s capability to capture full-length transcripts.

#### Gene ontology enrichment analysis

Nanopore sequencing data files processed with FeatureCounts were merged into a single dataset to create gene expression count tables. Phenotypic data were matched to the count data by sample identifiers for downstream analysis. Spearman’s correlations were calculated between gene expression and a chosen phenotype. Genes were ranked based on the strength of their correlation, and enrichment analysis was performed using Gene Set Enrichment Analysis to identify significantly associated GO terms (Biological Process) (*p* ≤ 0.05).^39^ Redundant GO terms were removed using the simplify function using the Wang method with a cutoff of 0.5. Top enriched terms were sorted on adjusted *p*-value and visualized as bar plots of normalized enrichment scores (NES) to highlight important biological processes.

### Quantification and statistical analysis

#### Statistical software and general approach

All statistical analyses were performed in R under RStudio build 2024.04.1+748. Continuous variables were summarized as mean and standard deviation when normally distributed and as median and interquartile range when not. Normality was assessed with the Shapiro-Wilk test. Two-group comparisons of continuous variables used Student’s *t* test for normally distributed data and the Wilcoxon Rank-Sum test otherwise. Categorical variables were compared with Fisher’s exact test due to small per-group sample sizes. Statistical significance was defined as *p* < 0.05 for unadjusted comparisons and adjusted *p* < 0.05 where Benjamini-Hochberg correction was applied. Correlations between continuous variables were computed using Pearson’s coefficient for normally distributed pairs and Spearman’s rank coefficient otherwise.

#### Mixed-model analysis of nuclear and vascular morphology

Nuclear morphology parameters (area, eccentricity, and solidity) were analyzed using linear mixed models with disease group as a fixed effect and cat identifier as a random intercept. The model family was selected by the Akaike information criterion (AIC), which is reported in Table S3. Pairwise contrasts were obtained with the emmeans package and adjusted for multiple testing with the Benjamini-Hochberg method.

Vessel parameters were compared across disease groups within each size class (small: <100 μm, medium: 100–250 μm, large >250 μm) using Kruskal-Wallis tests. Where the global test was significant, pairwise comparisons were performed using log-Gaussian mixed models with Benjamini-Hochberg adjustment.

#### Sample size and blinding

Sample size was defined by the available cohort. Slides were analyzed in QuPath under coded identifiers, blinding the operator to group assignment throughout image analysis. No data points were excluded on the basis of their results.

## Supplemental information

Document S1. Figures S1–S3, Data S1, and Tables S1 and S2

Table S3. Complete results of all statistical analyses across outcome groups, related to STAR Methods Summary of statistical analyses performed. Methods used include parametric and non-parametric tests, with results presented as test statistics, *p* values, confidence intervals, and normality assessments where applicable. Abbreviations: AIC, Akaike information criterion; ATE, arterial thromboembolism; BH, Benjamini-Hochberg; CI, confidence interval; df, degrees of freedom; EMM, estimated marginal mean; LMM, linear mixed model; LD, lumen diameter; MAE, mean absolute error; *MYH6*, myosin heavy chain 6; *MYH7*, myosin heavy chain 7; *NPPA*, natriuretic peptide A; OD, outer diameter; qPCR, quantitative polymerase chain reaction; RMSE, root-mean-square error; SE, standard error; WCSA, wall cross-sectional area; WLR, wall-to-lumen ratio; WT, wall thickness

Table S4. Pairwise correlations among demographic, histological, and clinical phenotype variables, related to Figure 4 Correlation analyses between phenotypic variables, including demographic, histological, and clinical data. Results are presented as correlation coefficients, raw and adjusted *p* values, confidence intervals, and the test method (Pearson or Spearman)

Table S5. Top 10 gene ontology biological process pathways from gene set enrichment analysis for each phenotype parameter, related to Figure 5 Gene set enrichment analysis (GSEA) results for genes correlated with different phenotype parameters. The table includes gene ontology (GO) terms (ID and description), set size, enrichment score, normalized enrichment score (NES), *p* value, adjusted *p* value, *q* value, rank, leading edge details, and core enrichment genes contributing to the enrichment
