# Supplementary material for: Phenotypic patterns in feline heart failure: A natural model for understanding variable disease severity in humans
Source: iScience. 2026 Jul 10;29(8):116685. doi: 10.1016/j.isci.2026.116685 (PMC13380742; doi:10.1016/j.isci.2026.116685)
Supplement: Document S1. Figures S1–S3, Data S1, and Tables S1 and S2 [file mmc1.pdf]

iScience, Volume *29*

Supplemental information

Phenotypic patterns in feline heart failure:

A natural model for understanding

variable disease severity in humans

Talitha C.F. Spanjersberg, Alma H. Hulsman, Guy C.M. Grinwis, Babette Janssen, C. Nina van der Wilt, Rogier J.A. Veltrop, Christian J.B. Snijders Blok, Claudia Rozendom, Paul J. Besseling, Jolanda van der Velden, Pim van der Harst, Magdalena Harakalova, and Frank G. van Steenbeek

SUPPLEMENTAL FIGURES

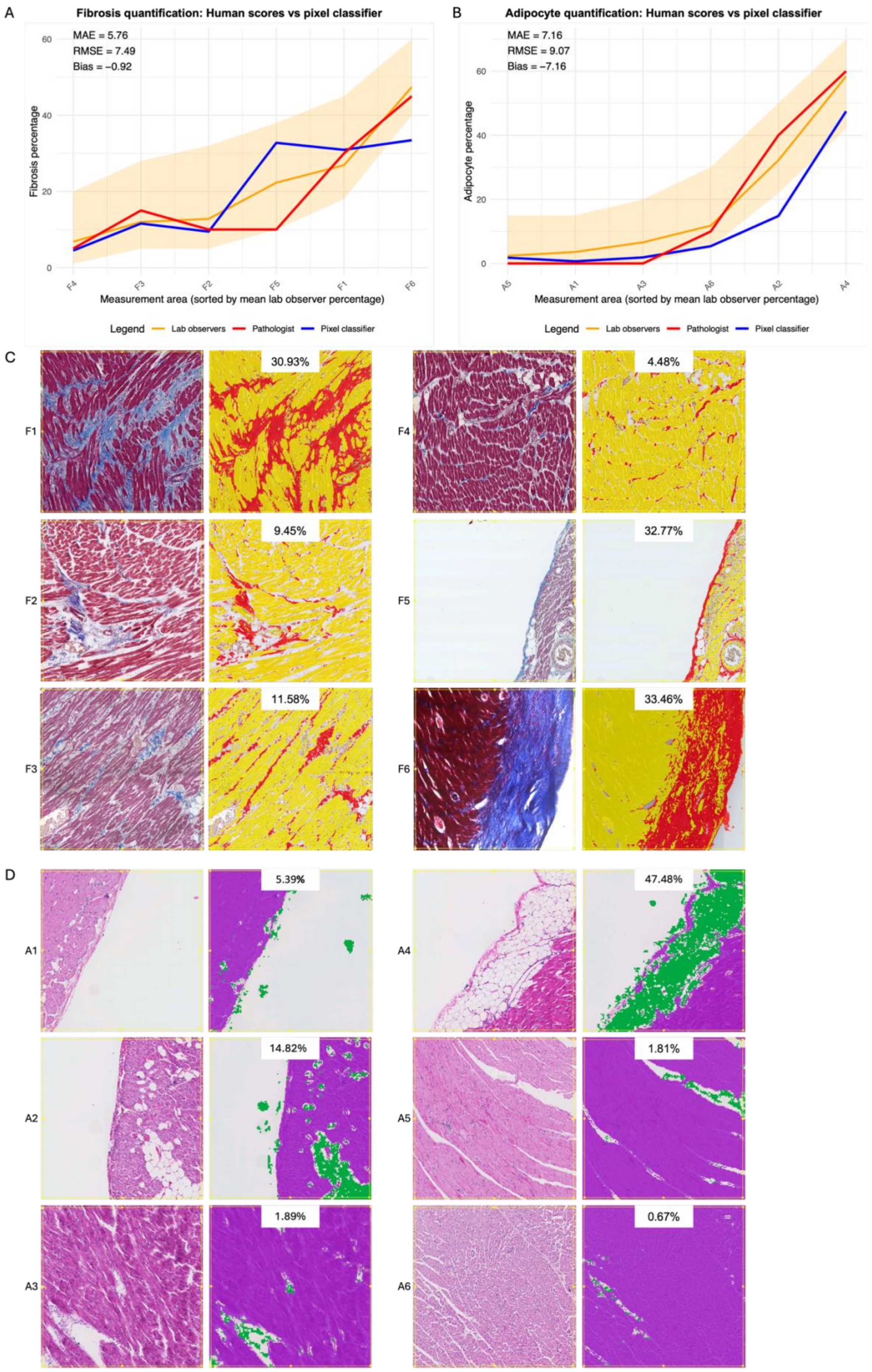
A
Fibrosis quantification: Human scores vs pixel classifier
MAE = 5.76
RMSE = 7.49
Bias = −0.92
Fibrosis percentage
Measurement area (sorted by mean lab observer percentage)
F4 F3 F2 F5 F1 F6
Legend Lab observers Pathologist Pixel classifier
B
Adipocyte quantification: Human scores vs pixel classifier
MAE = 7.16
RMSE = 9.07
Bias = −7.16
Adipocyte percentage
Measurement area (sorted by mean lab observer percentage)
A5 A1 A3 A6 A2 A4
Legend Lab observers Pathologist Pixel classifier
C
F1 30.93%
F4 4.48%
F2 9.45%
F5 32.77%
F3 11.58%
F6 33.46%
D
A1 5.39%
A4 47.48%
A2 14.82%
A5 1.81%
A3 1.89%
A6 0.67%

Figure S1: Validation of pixel classifier for fibrosis and adipocyte quantification
(A) Fibrosis quantification: Human scores vs pixel classifier. Line plot comparing fibrosis percentages quantified by lab observers (orange), a pathologist (red), and the pixel classifier (blue) for six measurement areas, sorted by increasing fibrosis percentage. The shaded area represents the range of human scores. The fibrosis classifier showed good agreement with human scoring, with a mean absolute error (MAE) of 5.76%, a root mean square error (RMSE) of 7.49%, and a small negative bias of -0.92%.

(B) Adipocyte quantification: Human scores vs pixel classifier. Line plot comparing adipocyte percentages determined by human scoring (orange) and the pixel classifier (blue) for six measurement areas, sorted by increasing adipocyte percentage. The shaded area represents the range of human scores. The adipocyte classifier captured the relative differences in adipocyte content across regions, with an MAE of 7.16% and an RMSE of 9.07%, and showed a negative bias of -7.16%, indicating lower estimates than the mean lab observer score.

(C) Randomly selected areas used for fibrosis validation. Percentages reflect the measurement of the pixel classifier. **(D)** Randomly selected areas used for adipocyte validation. Percentages reflect the measurement of the pixel classifier.

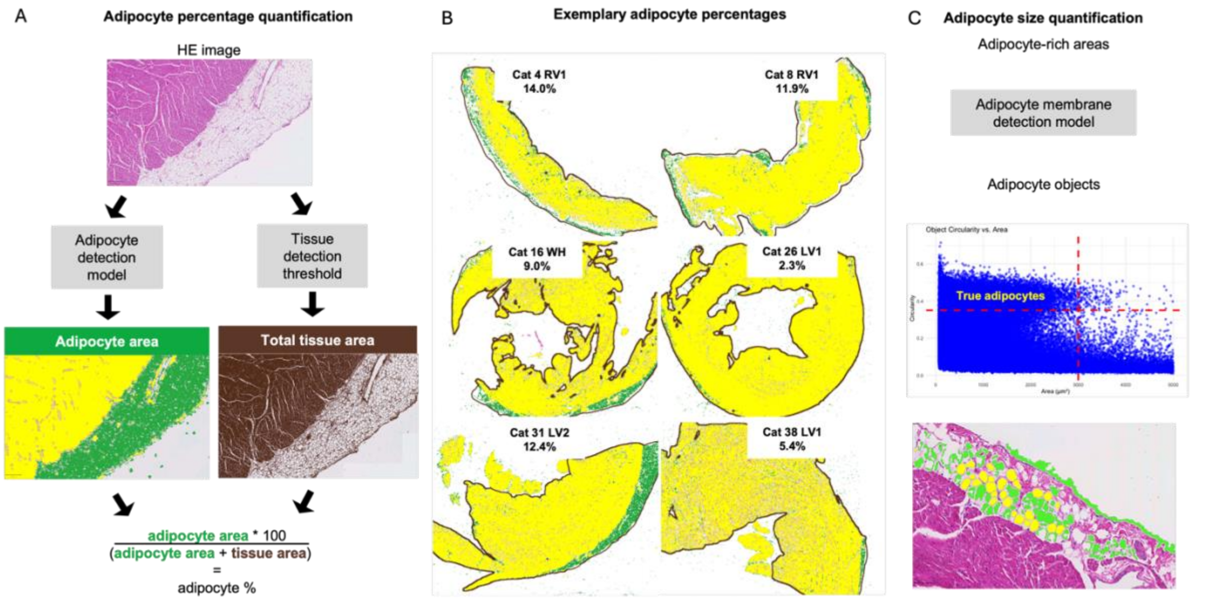

Figure S2: Adipocyte quantification

(A) Adipocyte percentage quantification. An HE-stained image (top) is processed using an adipocyte detection model (left) to identify adipocyte areas (green) and a tissue detection threshold (right) to determine the total tissue area (brown). Adipocyte percentage is calculated using these values. **(B)** Exemplary adipocyte percentages. Representative examples of cardiac sections from different cats with varying adipocyte percentages. The detected adipocyte areas (green) are overlaid on the myocardial tissue (yellow). **(C)** Adipocyte size quantification. Adipocyte-rich areas are analyzed using an adipocyte membrane detection model to identify individual adipocyte objects. Features such as area and circularity are calculated for each object. The scatter plot (top) distinguishes true adipocytes from rejected objects using strict filtering thresholds. The bottom panel shows a visual example where rejected objects and true adipocytes are overlaid on the histological image.

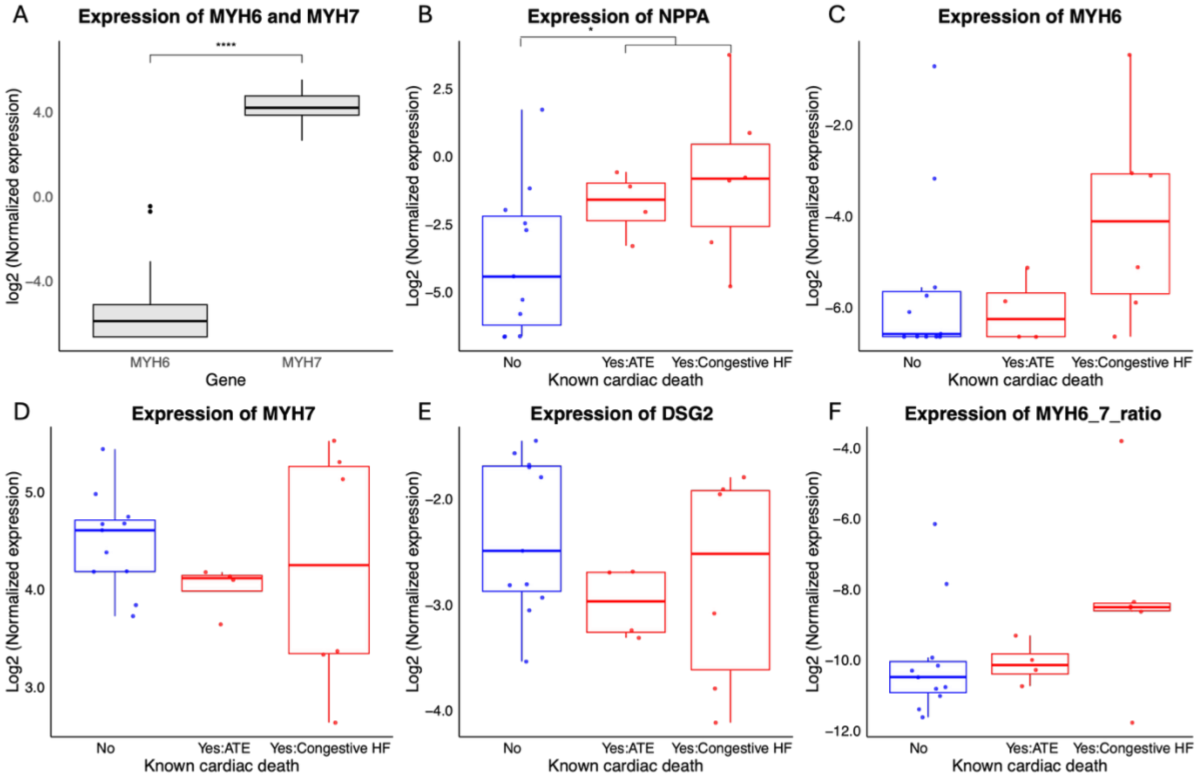

Figure S3: Gene expression analysis using qPCR. This figure presents the log2 normalized expression of selected genes in ventricular samples of 21 cats. *MYH7* shows significantly higher expression compared to *MYH6* (**** $p<0.0001$) in all samples. *NPPA* expression is significantly higher in cats with known cardiac death (* $p<0.05$). The expression of *MYH6*, *MYH7*, *DSG2,* and the ratio of *MYH6*:*MYH7* did not significantly change in association with known cardiac death or between the outcome groups.

DATA S1: Adipocyte quantification and qPCR for molecular markers

Adipocyte quantification

At higher resolutions, the model for quantifying adipocytes frequently misclassified artifacts such as tissue tears and vessel lumens as adipose tissue. To enhance accuracy, we adjusted the classifier to operate at a lower resolution, which minimized misclassifications and improved the reliability of our results (**Figure S2A**). The model achieved a MAE of 7.16% and a RMSE of 9.07% compared to the human mean. While these error metrics were slightly higher than the average variability among human observers (MAE: 5.46%, RMSE: 6.37%), the pixel classification model consistently estimated lower adipocyte percentages across regions (**Figure S1B**). The consistent tendency toward lower scores suggests model stability, though further validation is needed to assess accuracy. Statistical analysis showed no significant difference between the pixel classification predictions and human scores (Mann-Whitney U; $p=4.41 \times 10^{-1}$), indicating general alignment with human evaluations despite the systematic bias toward lower scores.

Adipocytes were predominantly located in the epicardial region and occasionally in the endocardial area (**Figure S2B**). Infiltration of adipocytes into the myocardium was mainly observed in the right ventricle. Adipocyte surface percentage was significantly higher in the right ventricle compared to the rest of the heart (median 7.66% vs. 4.35%; WSRT; $p=1.02 \times 10^{-7}$; n=30). Across the entire cohort, the adipocyte surface percentage ranged from 2.11-12.01% with a mean of 5.22%. No significant difference in adipocyte percentage was found between cats in the different outcome groups (**Table S3**). To quantify adipocyte size, we adapted a previously published method (**Figure S2C**)[35]. Due to tissue tears and disruptions in adipocyte membranes, many detected objects were erroneously identified as adipocytes. Therefore, we plotted object size against circularity and observed a distinct cluster of round objects within the expected size range for adipocytes. By applying strict filtering criteria based on size and circularity, we selected only those objects that could be confidently classified as adipocytes. After visual inspection, adipocyte size measurements from cats with very low adipocyte percentages (<3%) were found to be unreliable due to artifacts such as fragmented cells and staining inconsistencies. Consequently, these data were excluded from the analysis, resulting in a final sample size of 29 cats for adipocyte size quantification. No significant difference in adipocyte size was found between cats in the different outcome groups (**Table S3**).

Age exhibited a strong negative correlation with mean adipocyte size ($r = -0.65$, Pearson; $p_{adj} = 6.22\times10^{-3}$), suggesting smaller adipocytes in older cats. Body condition score showed a strong positive correlation with adipocyte size ($r = 0.52$, Spearman; $p_{adj} = 7.62\times10^{-2}$), suggesting that animals with higher body condition scores tend to have larger adipocytes. However, adipocyte percentage was not significantly correlated with body condition ($r = 0.043$, Spearman; $p_{adj} = 9.25\times10^{-1}$) (**Table S4**). Adipocyte size was also strongly correlated with nuclear eccentricity in longitudinal sections ($r = 0.51$, Pearson; $p_{adj} = 3.50\times10^{-2}$). Adipocyte percentage showed a significant negative correlation with nucleus size in both the transversal ($r = -0.57$, Spearman; $p_{adj} = 6.22\times10^{-3}$) and longitudinal ($r = -0.49$, Spearman; $p_{adj} = 2.53\text{x}10^{-2}$) sections.

qPCR for molecular markers

To investigate the expression of key genes involved in heart failure, we performed RT-qPCR analyses of *MYH6*, *MYH7*, *DSG2*, and *NPPA* on left ventricular samples from 21 cats. The mean normalized log2 expression levels were -5.23±3.81 for *MYH6* and 4.32±0.56 for MYH7, demonstrating a significantly higher expression of *MYH7* compared to *MYH6* (paired t-Test, $p=9.14\times10^{-16}$; CI: 8.71-10.42; n=21, **Figure S3**). When comparing gene expression between cats with a known cardiac death and those without, *NPPA* expression was significantly higher in the cardiac death group (-1.21 vs. -3.83; independent t-Test, $p=3.12\times10^{-2}$; CI: -4.97 to -0.26; n=21). In contrast, no significant differences were observed in the expression levels of *MYH6*, *MYH7*, or *DSG2* between the two groups or three groups nor between the expression levels of *MYH6*, *MYH7*, *DSG2*, or *NPPA* and the percentage of myocardial fibrosis (**Table S3**).

References:

35. Palomäki, V.A., Koivukangas, V., Meriläinen, S., Lehenkari, P., and Karttunen, T.J. (2022). A Straightforward Method for Adipocyte Size and Count Analysis Using Open-source Software QuPath. Adipocyte *11*, 99–107.

Table S1: Segmentation performance metrics for the Cellpose nucleus segmentation model, related to STAR Methods.

Performance metrics of the final Cellpose segmentation model after two training rounds, including Intersection over Union (IoU), precision, recall, accuracy, F1-score, and Panoptic Quality. Abbreviations: n_true: Ground truth nuclei count; n_pred: Predicted nuclei count; mean_matched_score: Mean IoU of matched true positives; mean_true_score: Mean IoU of matched true positives, normalized by the total number of ground truth objects; panoptic_quality: Segmentation and classification performance.

Region	Prediction v. GT Intersection over Union	false positive	true positive	false negative	precision	recall	accuracy	f1 score	n_true	n_pred	mean_true_score	mean_matched_score	panoptic_quality
1	0.736194947	0	14	3	1	0.82352941	0.82352941	0.90322581	17	14	0.64524332	0.783509746	0.707686222
2	0.707427233	0	16	3	1	0.84210526	0.84210526	0.91428571	19	16	0.681047463	0.808743863	0.73942296
3	0.76427379	2	14	6	0.875	0.7	0.63636364	0.77777778	20	16	0.602136779	0.860195399	0.669040866
4	0.816474343	0	16	2	1	0.88888889	0.88888889	0.94117647	18	16	0.728346732	0.819390074	0.771190658
5	0.806163828	0	5	1	1	0.83333333	0.83333333	0.90909091	6	5	0.750108508	0.90013021	0.818300191
6	0.849552724	0	13	1	1	0.92857143	0.92857143	0.96296296	14	13	0.784209324	0.844533119	0.813254114
7	0.642824181	0	19	8	1	0.7037037	0.7037037	0.82608696	27	19	0.550964193	0.782949117	0.646784053
8	0.680661754	0	12	2	1	0.85714286	0.85714286	0.92307692	14	12	0.700078286	0.816758	0.753930462
9	0.762009213	1	30	11	0.96774194	0.73170732	0.71428571	0.83333333	41	31	0.598993502	0.818624453	0.682187044

Table S2: Forward and reverse primer sequences and annealing temperatures for RT-qPCR, related to STAR Methods.

Sequences of forward and reverse primers used for gene amplification, with their corresponding annealing temperatures (Temp, °C). Abbreviations: NPPA: natriuretic peptide A; MYH6: myosin heavy chain 6; MYH7: myosin heavy chain 7; DSG2: desmoglein 2; RPS5: ribosomal Protein S5; HPRT-1: hypoxanthine phosphoribosyltransferase 1; YWHAZ: tyrosine 3-monooxygenase/tryptophan 5-monooxygenase activation protein zeta.

Gene	Forward Primer (5'-3')	Reverse Primer (5'-3')	Temp (°C)
NPPA	TTC CTC CTC TTC CTG GCG T	GGG CAC GAC TTC ATC TTC TAA AGG	58.8 65
MYH6	AAT GAC AAC TCC TCC CGC T	ATC ACC CGA GAC TTC TCC AG	61.1
MYH7	GCT GAT CAC CAA CAA CCC	TCC TCT GAA GTG AAG CCC	61.1 59.8 64.6
DSG2	CTT TGT TGG GTC TGT TGA AGA G	TAG AAC ACC GGA GGA TAA GCA	64.6 62.7
RPS5	CAG GTC TTG GTG AAT GCG	CCA GAT GGC CTG ATT CAC	55-63
HPRT-1	TTA TGG ACA GGA CCG AGC	GTC AGC AAA GAA TTT ATA GCC C	60
YWHAZ	GAA GAG TCC TAC AAA GAC AGC ACG C	AAT TTT CCC CTC CTT CTC CTG C	65
